# Predictors of treatment outcomes in group cognitive behavioural therapy for orphaned adolescents in sheltered homes

**DOI:** 10.3389/fpsyt.2026.1747075

**Published:** 2026-07-31

**Authors:** Wai Eng Ding, Firdaus Mukhtar

**Affiliations:** 1 Ding Psychology Centre, Petaling Jaya, Selangor, Malaysia; 2 Department of Medicine & Health Sciences, Universiti Putra Malaysia, UPM Serdang, Serdang, Selangor, Malaysia

**Keywords:** adolescents, anxiety, cognitive behaviour therapy, depression, predictor

## Abstract

**Introduction:**

Research on predictors of group cognitive behaviour therapy (gCBT) outcomes in adolescents with depression and anxiety is limited. This study aims to analyse factors predicting outcomes of a gCBT intervention targeting depression and anxiety in orphaned adolescents living in sheltered homes in Selangor, Malaysia. It examines the predictive value of sociodemographics, intervention type (gCBT versus waitlist control), and pre-intervention scores of psychological variables for immediate post-intervention and pre-post intervention change scores.

**Methods:**

Using cluster sampling, 17 sheltered homes were identified, and nine participated in the study. These homes were randomly assigned to either the gCBT or waitlist control group. Participants in the intervention group attended eight sessions of the gCBT (SAHABAT program) over one month. Data were collected at pre-intervention, mid-intervention, and immediate post-intervention using self-report questionnaires. Scores were analysed using a generalised linear mixed model.

**Results:**

Intervention type emerged as a significant predictor of outcomes for both depression and anxiety at immediate post-intervention, as well as for pre-post intervention change scores, indicating the gCBT was more effective than the waitlist control. At the immediate post-intervention, pre-intervention depression scores significantly predicted depression outcomes, while pre-intervention anxiety scores significantly predicted anxiety outcomes. Specifically, lower pre-intervention depression and anxiety scores, along with higher self-esteem scores, predicted better intervention outcomes. Pre-intervention scores of negative automatic thoughts predicted depression outcomes only. Additionally, pre-intervention anxiety scores influenced the pre-post change score for depression, whereas the pre-intervention depression scores did not affect the pre-post change score for anxiety. Age was also a significant predictor.

**Conclusion:**

The results and implications of these findings are discussed.

## 1 Introduction

Adolescent mental health in Malaysia is a growing public health concern, with studies reporting relatively high prevalence of depressive symptoms (17%–27%) and anxiety (10%–25%), particularly among vulnerable and institutionalised populations where rates are significantly higher (1–3). Concurrently, Malaysia has a substantial number of children in nongovernment-run institutional care, estimated at 64,000, with most coming from disadvantaged family backgrounds rather than orphanhood (4). Limited formal care capacity and constrained opportunities for adoption further reflect systemic challenges in child welfare services (5). Given the overlap between family vulnerability, institutionalisation, and elevated mental health risks, there is a need to better understand and address the psychosocial needs of adolescents within these contexts.

Literature reviews showed that age and gender were not associated with CBT treatment outcomes for both depression and anxiety in children and adolescents (6, 7). Nilsen et al. (7) reported that most studies showed that sociodemographic variables seldom predicted CBT treatment outcomes for anxiety and depression. However, Nilsen et al. (7) indicated that while ethnicity did not influence CBT treatment outcomes for anxiety, ethnicity interfered with CBT treatment for depression, especially in ethnic minority groups.

Baseline scores of psychological factors, and other factors such as familial and environmental factors, and some common factors tended to have stronger predictive abilities than sociodemographic factors for CBT treatment outcomes of depression and anxiety. Often, depression was a strong predictor of anxiety in a high-risk sample of adolescents and vice versa (8, 9), depending on the baseline severity. Nilsen et al. (7) demonstrated that baseline symptom severity and comorbidity of depression and anxiety were predictive of CBT outcomes for depression, but not for anxiety. On the other hand, Lee et al. (10) showed that low baseline anxiety severity and higher global functioning predicted better CBT treatment outcomes for anxiety. However, Nilsen et al. (7) emphasised the need for further evidence to conclusively establish how internalizing comorbidity within anxiety disorders contributes to poorer treatment outcomes for depression.

Other psychological factors, familial and environmental factors, treatment modalities, and certain common factors tended to predict CBT treatment outcomes for depression and anxiety. Adolescents were more likely to achieve remission with medication alone or combined with CBT when they entered treatment with lower depression severity and fewer cognitive, anxiety, and family-related risk factors, particularly in the absence of comorbid dysthymia (11). Clinical features of anxiety (e.g., higher severity, duration, and avoidance), as well as comorbidity with other mental disorders, are particularly useful for predicting an unfavourable course of anxiety (12).

Negative self-talk has been associated with poorer CBT treatment outcomes for anxiety (13). Cognitive variables such as high rational thoughts, low hopelessness, negative thoughts, cognitive distortions (14), and hopelessness and suicidal ideation (11) are significant predictors of positive treatment outcomes of CBT in depressed adolescents. On the other hand, cognitive variables such as a decrease in negative automatic thoughts and an increase in anxiety control predicted the treatment outcome of CBT for anxious adolescents (15).

Studies on the predictive abilities of self-esteem and anger on the CBT treatment outcomes for depression and anxiety in adolescents are limited. Nevertheless, dysfunctional anger was associated with and predicted a variety of other adverse mental and physical health illnesses (such as depression, cardiovascular disease, and pain disorder), interpersonal conflict (such as parent-child conflict and marital conflict), and aggression (such as domestic violence and physical damage) (16). In addition, Waite et al. (17) found that low self-esteem predicted poorer psychotherapy outcomes for psychiatric disorders, including depression.

Lastly, most of the findings showed that treatment types (e.g., individual, group, and self-help) (18), adherence to the number of sessions attended, and homework completion (10) did not predict the CBT outcome for anxiety.

Overall, studies on pre-intervention patient variables such as depression, anxiety, negative automatic thoughts, self-esteem, and anger as predictors of CBT intervention outcomes for depression and anxiety are still scarce, particularly in the Asian orphan population. This study aims to fill this gap by examining predictors of gCBT intervention outcomes for depression and anxiety at immediate post-intervention outcomes, and changes in scores from pre- to post-intervention, among orphans in Malay-operated nongovernment-run sheltered homes in Selangor, Malaysia. Factors considered include socio-demographic characteristics (such as age, gender, type of orphan, history of counselling attendance), intervention type (i.e., gCBT versus waitlist control), and pre-intervention scores of depression, anxiety, negative automatic thoughts, self-esteem, and anger.

## 2 Materials and methods

### 2.1 Sampling

The sampling frame was obtained from various official sources, including the Registrar of Societies of Malaysia, the Department of Social Welfare Malaysia, and the Department of Islamic Selangor, to ensure representation and exclude potential biases. Selangor state was chosen as the study site because it provides access to a substantial number of registered sheltered homes, facilitating the recruitment of a larger participant sample. Furthermore, adolescents in Selangor have been reported to experience relatively higher rates of depression and anxiety compared to those in other Malaysian states (3, 19, 20).

The study included Malay-operated, nongovernment-run sheltered homes that supported underprivileged orphaned and non-orphaned children and adolescents by providing housing, meals, financial aid, healthcare access, and enrolment in national schools. Several types of facilities were excluded from participation, including government-operated sheltered homes and non-Malay-operated sheltered homes, religious and homeschooling institutions, and administrative centres without residential services. Homes with fewer than three adolescent residents, facilities serving adults or elderly individuals, and centres catering to persons with physical or intellectual disabilities were also excluded. Additionally, kindergartens, childcare facilities, nursing homes, and community service centres were not considered eligible for the study. This study excluded government-run sheltered homes to ensure consistency and large sample sizes, and focused specifically on Malay-operated non-government-run homes to consider cultural and linguistic factors.

Using multistage cluster sampling, 17 Malay-operated, nongovernment-run sheltered homes that met the eligibility criteria were identified, of which nine agreed to participate in the study. These homes were then randomly assigned to either the gCBT intervention group or the waitlist control group, with four homes allocated to the gCBT group and five homes to the waitlist control group.

The Accuracy in Parameter Estimation (AIPE) method was employed to improve the precision of population parameter estimates by ensuring adequate sample sizes and appropriately narrow confidence intervals.

### 2.2 Participants

Both the administrators and adolescents were informed about the study objectives, procedures, and duration. Adolescents who met the eligibility requirements were invited to participate on a voluntary basis after being informed about the study aims, participation requirements, and their right to discontinue involvement at any stage. Consent for participation was provided by the administrators, who served as the adolescents’ legal guardians.

Inclusion criteria for participants were orphaned adolescents aged 13–17 years, who showed symptoms of depression and/or anxiety (BDI-Malay ≥ 10; BAI-Malay ≥ 8), had stayed in the sheltered home for a minimum of one day, and could communicate proficiently in the Malay language. Adolescents were excluded if they were non-orphans, had a learning disability or organic brain disorder, were currently prescribed psychotropic medication, undergoing psychotherapy, or were unavailable during the recruitment phase. The selected age range of 13–17 years was intended to ensure participants possessed sufficient cognitive and language abilities to engage effectively in cognitive behavioural therapy (CBT) (21). Although variables such as parental loss and resilience may contribute to emotional distress, these factors could not be comprehensively examined due to practical limitations. All identified sheltered homes housed both orphans and non-orphans. Non-orphans were not included because the study funding was specifically intended for interventions addressing the well-being of orphaned adolescents. Participants in the waitlist control group and non-Malay operated nongovernmental-run sheltered homes who continued to meet the study eligibility criteria were offered the same intervention after the completion of the study in accordance with ethical research practices, ensuring that they were not denied access to a potentially beneficial psychological intervention.

**Figure 1:**
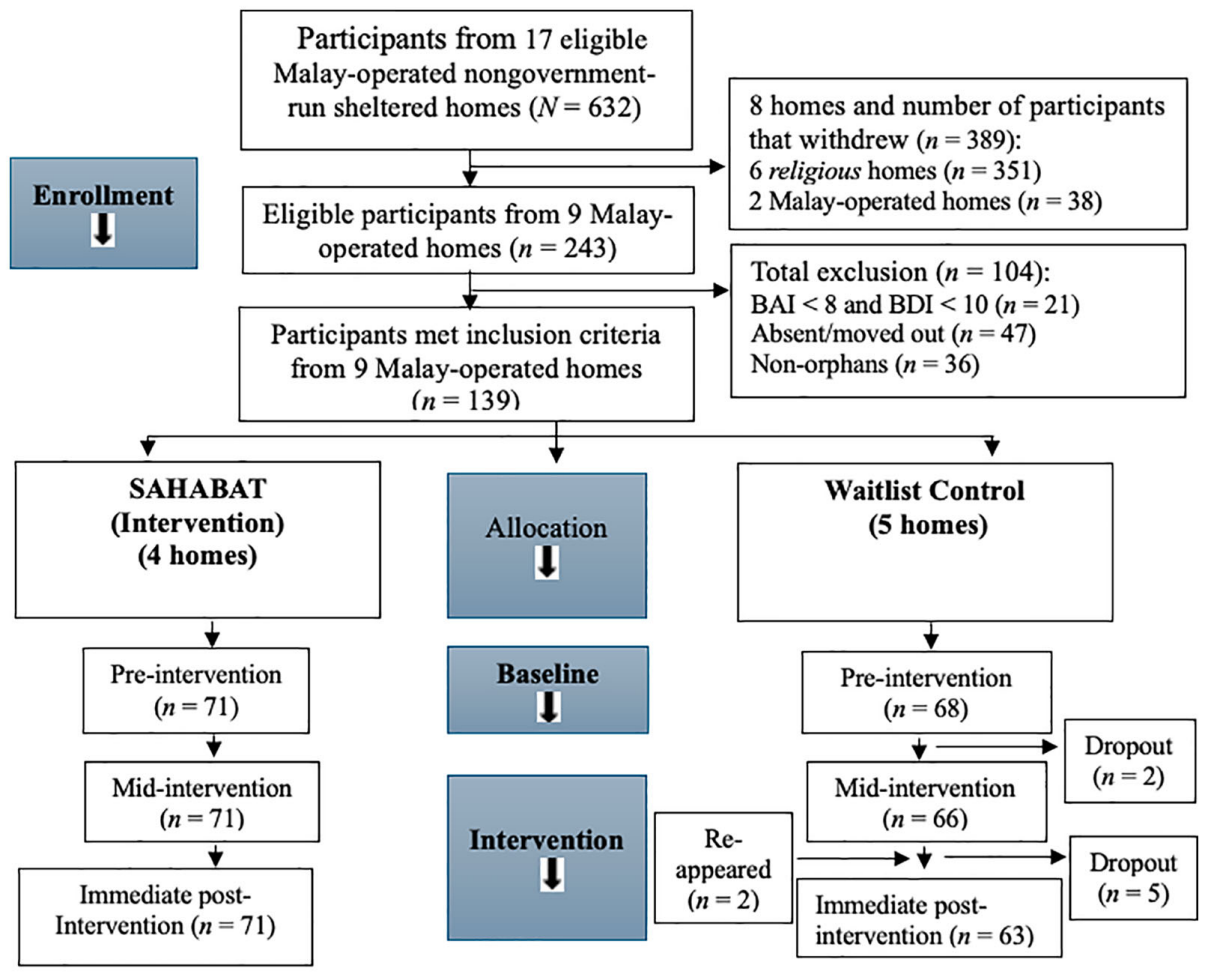
Consort flow chart.

**Figure 1** presents the CONSORT flow diagram. Initially, 17 Malay-operated, nongovernment- run sheltered homes (N = 632 adolescents) were randomised. Of the 632 potential participants, only 243 adolescents from the nine consenting homes were eligible for further screening. Among these, 139 orphaned adolescents met the inclusion criteria and were retained in the final sample, with 71 allocated to the intervention group and 68 to the waitlist control group.

The slight variation in group size was attributable to the cluster randomisation approach, in which all adolescents within the same sheltered home were assigned to a single study condition.

### 2.3 Procedure

#### 2.3.1 Pilot study

A preliminary pilot study was conducted to evaluate the feasibility, acceptability, and potential effectiveness of the SAHABAT programme, as well as its suitability for replication in similar settings (22).

#### 2.3.2 SAHABAT program

The efficacy of the gCBT was tested in addressing depression and anxiety among 139 orphaned adolescents aged 13–17 years (gCBT, *n* = 71; waitlist control, *n* = 68) in Malay-operated nongovernment-run sheltered homes in Selangor. The gCBT manual, SAHABAT or its English translation, FRIENDS (23) — was written in Malay and consists of eight core sessions as follows: Session 1 focused on ice-breaking activities, personal perceptions, and self- evaluation; Session 2 introduced relaxation strategies, self-esteem building, and anger management; Session 3 addressed the recognition of physiological symptoms, cognitive distortions, emotions, and behaviours; Session 4 emphasised strategies for modifying cognitive distortions to facilitate emotional and behavioural change; Session 5 covered problem-solving skills; Session 6 identified own support system; Session 7 focused on cultivating inner peace through positive self-talk; and Session 8 involved a review and consolidation of learning, culminating in a celebration of participants’ progress.

The intervention was delivered by the first author, who is an experienced, registered clinical psychologist. Participants in the intervention group attended eight sessions of the gCBT over one month. The SAHABAT is developed by the second author, who is a registered clinical psychologist and a professor at Universiti Pertanian Malaysia. The original SAHABAT manual was implemented in its entirety. There were no modifications were introduced following the pilot study, including no changes to the number of sessions, session content, delivery procedures, and participant materials. The waitlist control group did not receive the intervention. After this study had ended, participants who were excluded from this study received a similar intervention provided by the first author.

### 2.4 Measures

Participants completed validated Malay-language versions of the following self-report questionnaires: The Malay versions of the Beck Depression Inventory (BDI-Malay) (24, 25) and the Beck Anxiety Inventory (BAI-Malay) (26, 27) both demonstrated strong construct validity and high internal consistency in Malay samples (α = .90). The Malay version of the Automatic Thoughts Questionnaire (ATQ-Malay) (28, 29) showed good factorial validity with Cronbach’s alphas ranging from.83 to.93. The Malay version of the Beck Youth Inventories–Anger Scale (BANI-Y Malay) (30, 31) demonstrated excellent reliability (α = .92) and convergent validity. The Malay version of the Rosenberg Self-Esteem Scale (RSES-Malay) (32, 33) also showed acceptable psychometric properties in Malay adolescents, with alphas around.80. The translated questionnaires retained the same scoring procedures, raw score ranges for severity classification, and interpretive guidelines as the original instruments.

### 2.5 Data collection

Data were collected at pre-intervention, mid-intervention, and immediate post-intervention using self-report questionnaires.

### 2.6 Data analyses

Preliminary analyses were conducted to ensure that the assumptions underlying advanced statistical analyses were satisfied. These included assessments of normality, linearity, multicollinearity, homoscedasticity, and the presence of outliers.

Several predictor variables were examined to identify factors predicting the effectiveness of the intervention on depression and anxiety outcomes. These comprised sociodemographic characteristics, including age, gender, orphan status, and history of counselling attendance; intervention type (gCBT versus waitlist control); and baseline psychological measures, including pre-intervention scores for depression, anxiety, negative automatic thoughts, self-esteem, and anger. The outcome variables included intervention outcome scores at immediate post-intervention for depression and anxiety, as well as pre-post intervention change scores (i.e., the difference between post-intervention score and pre-intervention score) for both depression and anxiety.

A Generalised Linear Mixed Model (GLMM) was employed to examine the predictors of intervention outcomes. For models assessing immediate post-intervention outcomes, the fixed effects included sociodemographic variables, intervention type, and baseline psychological scores. In models assessing pre-post intervention change scores, the corresponding baseline psychological variable was excluded from the fixed model to avoid statistical redundancy. Specifically, baseline depression scores were omitted from the depression change-score model, while baseline anxiety scores were also excluded from the anxiety change-score model.

When the dependent variable was the immediate post-intervention score, the fixed model included: (a) sociodemographic variables such as age, gender, type of orphan (i.e., single orphan and double orphan), and history of counselling attendance; (b) type of intervention (i.e., gCBT versus waitlist control); and (c) psychological variables such as pre-intervention scores of depression, anxiety, negative automatic thoughts, self-esteem, and anger. When the dependent variable was a pre-post intervention change score for depression, the fixed model variables were similar, including the sociodemographic variables, type of intervention, and psychological variables, except that the pre-intervention score of depression was excluded. Similarly, when the dependent variable was a pre-post intervention change score for anxiety, the fixed model variables included the sociodemographic variables, type of intervention, and psychological variables, except for the pre-intervention score of anxiety.

Model fit was evaluated using the Akaike Information Criterion (AIC), with smaller AIC values indicating superior model fit. In the random-effects component of the GLMM, sheltered homes were specified as both random intercepts and random slopes to account for clustering effects and variability across sheltered homes. An intraclass correlation (ICC) and a design effect were calculated. The ICC was computed using the formula ρ = σ^2B^/(σ^2B^ + σ^2w^), where σ^2B^ represented the intercept variance of the random effect between the homes (Level 2), while σ^2w^ represented the variance of the individuals within the sheltered homes (Level 1). The design effect was calculated as D_eff_ = 1 + (m – 1) ρ, whereby m denoted the number of participants per home and ρ was ICC. An ICC >.05 (34) and a design effect > 2 (35) indicated the need for the GLMM analysis; otherwise, repeated measures of ANOVA might be appropriate. A higher ICC value indicated a greater clustering and violation of the independence assumption, supporting the use of GLMM.

## 3 Results

### 3.1 Sociodemographic characteristics

**Table 1** presents the sociodemographic profile of orphaned adolescents participating in this study. The entire cohort consisted of Malay individuals (99.9%) who adhered to the Islamic faith. On average, participants were 14.81 years old (SD = 1.37). The majority (67.6%) fell within the younger adolescent bracket (13–15 years), while the remaining 32.4% were classified as older adolescents (16–17 years). Gender distribution was relatively balanced, with 54.7% being female and 45.3% male. Single orphans comprised the majority (91.4%), while double orphans constituted a smaller proportion (8.6%). Single orphan means the adolescents lost one of their parents, while double orphan means they lost both parents. Approximately half (54%) had previously undergone school-based counselling, while the remainder (46%) had not. Comparison of sociodemographic characteristics between the participants of gCBT and the waitlist control group at baseline, excluding the analysis of religious affiliation, ethnicity, and educational type due to near-universal representation as Malay Muslims attending public schools. Results indicated no significant disparities in age, gender, or orphan status between the gCBT and control groups at pre-intervention. Notably, although disparities in counselling history were observed, instances of attendance were primarily limited to one or two sessions, with the majority attending less than three times.

**Table 1:** Sociodemographic characteristics of orphaned adolescents at pre-intervention for gCBT and waitlist control (n = 139).

Socio-demographic characteristic	Total		gCBT (*n* = 71)		Waitlist (*n* = 68)		Total	Stat. test	*P*
	n	%	n	%	n	%	N		
Age (M = 14.81, SD = 1.37)									
Age group (years)									
13–15	94	67.6	44	46.8	50	53.2	94	χ^2^ = 1.62	.20
16–17	45	32.4	27	60.0	18	40.0	45		
Gender									
Female	76	54.7	43	56.6	33	43.4	76	χ^2^ = 1.57	.21
Male	63	45.3	28	44.4	35	55.6	63		
Type of orphan									
Single	127	91.4	66	52.0	61	8.0	127	χ^2^ = .15	.70
Double	12	8.6	5	41.7	7	58.3	12		
HoCA									
Attended	75	54.0	45	60.0	30	40.0	75	χ^2^ = 4.44	.04*
Never attended	64	46.0	26	40.6	38	59.4	64		

Significant at *p <.05 (2-tailed). HoCA, History of counselling attendance.

### 3.2 Pre-intervention

**Table 2** demonstrates that there are no statistically significant variances in depression, anxiety, negative automatic thoughts, self-esteem, and anger scores between the two groups, the gCBT and the waitlist control group, during the pre-intervention period.

**Table 2:** Depression, anxiety, negative automatic thoughts, self-esteem, and anger of orphaned adolescents at pre-intervention for gCBT and waitlist control (n = 139*).*

Measure	gCBT mean (*SD*) *n* = 71	Waitlist mean (*SD*) *n* = 68	Statistical test	*P*
BDI-Malay	17.73 (9.90)	17.97 (8.92)	*t* (137) = –.15	.88
BAI-Malay	23.44 (10.62)	20.16 (9.66)	*t* (137) = 1.90	.06
ATQ-Malay	39.28 (10.22)	35.68 (10.26)	*t* (137) = 1.88	.06
RSES-Malay	31.86 (3.71)	31.37 (3.72)	*t* (137) = .78	.44
BANI-Y Malay	22.65 (9.24)	19.29 (11.43)	*t* (137) = 1.91	.06

Significant at *p <.05 (2-tailed).

### 3.3 Predictors of intervention outcome for depression

**Table 3** shows that the grand mean of the total depression score at immediate post-intervention was 11.74 (SE = 1.39). The findings indicated that the type of intervention significantly predicted the immediate post-intervention depression score (β = –6.11, *t* = –2.27, *p* <.02, 95% CI [–11.40, –.82], η^2^ = .04). Participants in the gCBT group demonstrated lower depression scores at immediate post-intervention compared to those in the waitlist control group, despite a small effect size.

**Table 3:** Predictors of intervention outcome for depression at immediate post-intervention (n = 139).

Predictor	β	SE	*t*	*p*	95% CI		η^2^
					LL	UL	
M = 11.74, SE = 1.39 Sociodemographic							
Age	.39	.20	1.97	<.05*	.001	.78	.03
Gender	–.48	.49	–.98	.33	–1.14	.48	.01
Type of orphan	.54	.86	.64	.53	–1.14	2.22	.01
HoCA	.01	.51	.01	.99	–1.00	1.01	.00
Type of intervention							
Group	–6.11	2.70	–2.27	<.02*	–11.40	–.82	.04
Pre-intervention scores							
BDI-Malay	.66	.03	21.09	<.001**	.60	.72	.76
BAI-Malay	.27	.03	9.06	<.001**	.21	.33	.37
ATQ-Malay	–.06	.03	–2.03	<.04*	–.12	–.00	.03
RSES-Malay	.08	.08	1.08	.28	–.07	.23	.01
BANI-Y Malay	–.05	.03	–1.62	.11	–.10	.01	.02

Significant at **p <.001 (2-tailed), *p <.05 (2-tailed). HoCA, History of counselling attendance.

Among the psychological variables, the pre-intervention depression score emerged as a highly significant predictor of the immediate post-intervention depression score (β = .66, *t* = 21.09, *p* <.001, 95% CI [.60,.72], η^2^ = .76), indicating a large effect size. Similarly, the pre-intervention anxiety score significantly predicted the immediate post-intervention depression score (β = .27, *t* = 9.06, *p* <.001, 95% CI [.21,.33], η^2^ = .37), also reflecting a large practical effect. In addition, the pre-intervention negative automatic thoughts score significantly predicted the immediate post-intervention depression score (β = –.06, *t* = –2.03, *p* <.04, 95% CI [–.12, –.00], η^2^ = .03). However, the practical effect of negative automatic thoughts was comparatively smaller than that of pre-intervention depression and anxiety scores. The pre-intervention scores of self-esteem and anger did not significantly predict the immediate post-intervention depression score.

About sociodemographic variables, age significantly predicted immediate post-intervention depression scores (β = .39, *t* = 1.97, *p* <.05, 95% CI [.001,.78], η^2^ = .03), indicating a small practical effect. In contrast, gender, type of orphanhood, counselling attendance history, self-esteem, and anger were not significant predictors of the immediate post-intervention depression outcomes.

Overall, among all significant predictors, the type of intervention had the highest β value, which significantly contributed to the prediction of the post-intervention depression score, supporting the effectiveness of the gCBT programme relative to the waitlist control condition. The pre-intervention depression score produced the strongest predictive effect on the immediate post-intervention depression outcomes among the psychological variables, followed by the pre-intervention anxiety score. Other significant predictors, in descending order of predictive strength, were age and the pre-intervention negative automatic thoughts score.

**Table 4** shows that the grand mean of the pre-post intervention change score for depression was -5.98 (SE = 1.64). The findings indicated that the type of intervention significantly predicted the pre-post intervention change score for depression (β = –7.19, *t* = –2.26, *p* = .03, 95% CI [–13.44, –.93], η^2^ = .04). The results suggest that participants in the gCBT group demonstrated greater reductions in depression scores compared to those in the waitlist control group, despite the practical effect was small.

**Table 4:** Predictors of pre-post intervention change score for depression (n =139).

Predictors	β	*SE*	*t*	*p*	95% CI		η^2^
					LL	UL	
M = -5.98, SD = 1.64 Sociodemographic							
Age	.69	.22	3.13	<.01**	.26	1.12	.07
Gender	–.09	.54	–.17	.87	–1.15	.97	.00
Type of orphan	.24	.94	.26	.79	–1.61	2.09	.00
HoCA	–.43	.56	–.08	.44	–1.54	.67	.00
Type of intervention							
Group	–7.19	3.19	–2.26	.03*	–13.44	–.93	.04
Pre-intervention scores							
BAI-Malay	.20	.03	6.27	<.001***	.14	.26	.22
ATQ-Malay	–.12	.03	–3.68	<.001***	–.18	-.06	.09
RSES-Malay	.41	.08	5.36	<.001***	.26	.56	.17
BANI-Y Malay	–.05	.03	–1.60	.11	–.11	.01	.02

Significant at ***p < .001 (2-tailed), **p < .01 (2-tailed), *p < .05 (2-tailed). HoCA, History of counselling attendance.

Among the psychological variables, the pre-intervention anxiety score significantly predicted pre-post intervention change score for depression (β = .20, *t* = 6.27, *p* <.001, 95% CI [.14,.26], η^2^ = .22), indicating a large practical effect. The pre-intervention self-esteem score also significantly predicted pre-post intervention change score for depression (β = .41, *t* = 5.36, *p* <.001, 95% CI [.26,.56], η^2^ = .17), reflecting a relatively large effect size. In addition, the pre-intervention negative automatic thoughts score significantly predicted the pre-post intervention change score for depression (β = –.12, *t* = –3.68, *p* <.001, 95% CI [–.18, –.06], η^2^ = .09), indicating a medium effect size. The pre-intervention score of anger did not significantly predict the pre-post-intervention change scores for depression.

Regarding sociodemographic variables, age significantly predicted the pre-post intervention change score for depression (β = .69, *t* = 3.13, *p* <.01, 95% CI [.26, 1.12], η^2^ = .07), suggesting a medium practical effect. In contrast, gender, type of orphanhood, counselling attendance history, and anger were not significant predictors of the pre-post intervention change score for depression.

Overall, among all predictors examined, the type of intervention emerged as the strongest predictor, as reflected by the highest β value, demonstrating the superiority of the gCBT programme compared to the waitlist control condition. Other significant predictors of pre-post intervention score for depression, in descending order of predictive strength, included pre-intervention anxiety and self-esteem scores, and pre-intervention negative automatic thought score, respectively. Age was also a significant predictor.

### 3.4 Predictors of intervention outcome for anxiety

**Table 5** shows that the grand mean of the total anxiety score at immediate post-intervention was 16.91 (SE = 1.25). The results showed that the type of intervention (gCBT versus waitlist control) significantly predicted the immediate post-intervention score for anxiety (β = – 8.66, *t* = -3.72, *p* <.001, 95% CI [-13.24, -4.09], η^2^ = .09), indicating a medium effect size.

**Table 5:** Predictors of intervention outcome for anxiety at immediate post-intervention (n = 139).

Predictor	β	SE	*t*	*p*	95% CI		η^2^
					LL	UL	
M = 16.91, SE = 1.25 Sociodemographic							
Age	.73	.26	2.84	<.01*	.23	1.24	.04
Gender	–.04	.63	–.06	.95	–1.28	1.20	.00
Type of orphan	1.26	1.10	1.15	.25	–.90	3.41	.01
HoCA	1.21	.66	1.83	.07	–.09	2.51	.02
Type of intervention							
Group	–8.66	2.33	-3.72	<.001**	–13.24	– 4.09	.09
Pre-intervention scores							
BDI-Malay	.11	.04	2.79	<.01*	.03	.19	.05
BAI-Malay	.66	.04	17.17	<.001**	.59	.74	.68
ATQ-Malay	–.00	.04	–.08	.93	–.08	.08	.00
RSES-Malay	.42	.10	4.28	<.001**	.23	.62	.12
BANI-Y Malay	.01	.04	.28	.78	–.06	.08	.00

Significant at **p <.001 (2-tailed), *p <.01 (2-tailed). HoCA, History of counselling attendance.

Among the psychological variables, the pre-intervention depression score significantly predicted the immediate post-intervention score for anxiety (β = .11, *t* = 2.79, *p* <.01, 95% CI [.03,.19], η^2^ = .05), indicating a small effect size. The pre-intervention anxiety score also significantly predicted the immediate post-intervention score for anxiety (β = .66, *t* = 17.17, *p* <.001, 95% CI [.59 to.74], η^2^ = .68), with a large effect size. The pre-intervention self-esteem score significantly predicted the immediate post-intervention score for anxiety (β = .42, *t* = 4.28, *p* <.001, 95% CI [.23,.62], η^2^ = .12), indicating a medium effect. In contrast, pre-intervention negative automatic thoughts and anger scores did not significantly predict the immediate post-intervention score for anxiety.

Sociodemographic variables, specifically age (β = .73, *t* = 2.84, *p* <.01, 95% CI [.23,.1.24], η^2^ = .04), indicate a small practical effect in predicting immediate post-intervention score for anxiety. In addition, gender, type of orphanhood, and history of counselling attendance were not significant predictors of the immediate post-intervention score for anxiety.

The GLMM results show a borderline statistically significant effect (*p* = .07) for history of counselling attendance, but the confidence interval (−.09 to 2.51) crosses zero, indicating uncertainty in the direction and precision of the effect. The effect size was also small (η^2^ = 0.02), suggesting limited practical significance.

Overall, among all predictors examined for the immediate post-intervention outcomes for anxiety, the type of intervention emerged as the strongest predictor, as reflected by the highest β value, demonstrating the superiority of the gCBT programme compared to the waitlist control condition. Other significant predictors of the immediate post-intervention outcomes for anxiety, in descending order of predictive strength, included pre-intervention anxiety, self-esteem, and depression scores, respectively. Age also emerged as a significant predictor.

**Table 6** shows that the grand mean of pre-post intervention change score for anxiety was 3.98 (SE = 1.36). Type of intervention (gCBT versus waitlist control) significantly predicted the pre–post intervention change score for anxiety (β = 11.57, *t* = 4.61, *p* <.001, 95% CI [6.65, 16.49], η^2^ = .14). The findings suggest that participants in the gCBT group demonstrated significantly greater reductions in anxiety compared to those in the waitlist control group.

**Table 6:** Predictors of pre-post intervention change score for anxiety (n =139).

Predictors	β	SE	*t*	*p*	95% CI		η^2^
					LL	UL	
M = 3.98 (SE = 1.36) Sociodemographic							
Age	–1.26	.26	–4.77	*p* < .001*	–1.78	–.74	.14
Gender	.04	.66	.05	.96	–1.26	1.34	.00
Type of orphan	–.91	1.16	–.78	.44	–3.18	1.37	.00
HoCA	–.52	.70	–.75	.46	–1.88	.85	.00
Type of intervention							
Group	11.57	2.51	4.61	*p* <.001*	6.65	16.49	.14
Pre-post change score							
BDI-Malay	–.04	.04	–.95	.34	–.12	.04	.01
ATQ-Malay	.08	.04	1.96	.05	–.00	.15	.03
RSES-Malay	–.40	.10	–3.87	*p* <.001*	–.60	–.20	.10
BANI-Y Malay	.06	.06	1.73	.09	–.01	.14	.02

Significant at **p <.001 (2-tailed), *p <.01 (2-tailed). HoCA, History of counselling attendance.

The results further indicated that among the psychological variables, only the pre-intervention self-esteem score significantly predicted the pre-post intervention change score of anxiety (β = -.40, *t* = -3.87, *p* <.001, 95% CI [-.60, -.20], η^2^ = .10), indicating a medium effect size. The borderline significance of the pre-intervention negative automatic thoughts score predicted the pre-post intervention change score for anxiety (β = .08, *t* = 1.96, *p* = .05, 95% CI [-.00 to.15, η^2^ = .03), with a small effect size. The pre-intervention negative automatic thoughts score approached statistical significance (*p* = .05); however, the confidence interval indicated uncertainty around the estimate (95% CI [–.00,.15]). Furthermore, the effect size was small (η^2^ = .03), suggesting limited practical significance. The pre-intervention score of anger did not significantly predict the immediate pre-post intervention change score for anxiety.

Sociodemographic predictors, particularly age, significantly predicted the pre–post intervention change score for anxiety (β = –1.26, *t* = –4.77, *p* <.001, 95% CI [–1.78, –0.74], η^2^ = .14), with a large effect size. The findings indicate that age contributed substantially to changes in anxiety score following the intervention. In contrast, gender, type of orphanhood, and counselling attendance history did not significantly predict pre-post intervention change score for anxiety.

Overall, among all predictors examined, the type of intervention emerged as the strongest predictor, as indicated by the highest β value, demonstrating not only the superiority of the gCBT programme compared to the waitlist control condition, but also a large practical effect. Other significant predictors of the pre-post intervention change score for anxiety included pre-intervention self-esteem score and age. Additionally, pre-intervention negative automatic thought score might be a significant predictor.

## 4 Discussion

The type of intervention (gCBT versus waitlist control) significantly predicted intervention outcomes for depression and anxiety, both immediately post-intervention and in terms of pre-post intervention change scores in this study. It emerged as the most influential predictor among all other significant factors. Although this was a short-term predictor investigation, the randomised controlled trial included extended follow-up assessments at 1-month, 3-month, and 6-month post-intervention, with gCBT shown to remain effective to treat depression and anxiety up to 6 months after the intervention (22).

It is worth noting that gCBT typically shows small-to-medium effects for depression and medium-to-large effects for anxiety. This difference may be because gCBT for anxiety directly targets symptoms through structured techniques such as exposure and cognitive restructuring, whereas depression is more heterogeneous and influenced by broader cognitive, behavioural, and interpersonal factors, leading to more variable outcomes. Consequently, anxiety interventions often yield comparatively larger treatment effects (36).

Generally, the findings of this study also support the notion that the predictive abilities of most sociodemographic variables for depression and anxiety in adolescents often offer inconclusive evidence (6, 7, 18). These preliminary findings of sociodemographic predictors add to the scarce literature in Malaysia.

Specifically, age emerged as a significant predictor of intervention outcomes for both depression and anxiety in this study, affecting immediate post-intervention results and pre-post intervention change scores. This finding aligns with previous research on major depressive disorder in Malaysian adults (37). Notably, since this study focused on adolescents, age played a crucial role in predicting the intervention effectiveness of gCBT. Moreover, no significant distinction was found between early and late adolescents regarding depression and anxiety outcomes, indicating that both groups could benefit from gCBT. In this study, age showed a statistically significant association, and its predictive strength appeared consistent, suggesting it may be a stable or robust predictor across contexts. However, the present results alone are insufficient to support a firm generalisation.

Gender did not predict intervention outcomes for depression and anxiety immediately post-intervention or in pre-post intervention change scores in this study. Thus, no significant gender differences were found in depression and anxiety outcomes among orphans, indicating equal benefit from CBT for both females and males.

Next, this study found that a history of counselling attendance did not significantly predict intervention outcomes for depression and anxiety, both immediately post-intervention and in terms of pre-post intervention change scores. This suggests that the number of counselling sessions attended by adolescents at schools may have been insufficient or less effective.

This study found that whether an orphan was a single orphan or a double orphan did not significantly predict intervention outcomes for depression and anxiety, both immediately post-intervention and in terms of pre-post intervention change scores. Despite potential bias due to a higher number of single orphans in the gCBT, it is worth noting that both groups received the same support and facilities at the sheltered homes. Further research is needed to delve deeper into this aspect.

This study’s findings align with prior research indicating that baseline severity influences treatment response for depression and anxiety (10, 11, 18). Pre-intervention scores for depression, anxiety, and negative automatic thoughts significantly predicted depression intervention outcomes immediately post-intervention. Notably, similar findings were reported in previous studies where low baseline depression severity predicted depression recovery with CBT (11), and anxiety was linked to intervention response in depressed adolescents (38). Additionally, this study supports Becker et al., (14) conclusion that negative thoughts predict positive CBT outcomes in depressed adolescents. Despite mild depression scores, participants exhibited moderate anxiety and high negative automatic thoughts at baseline, underscoring the effectiveness of the gCBT in addressing depression. Moreover, the type of intervention emerged as the best predictor for depression intervention outcomes immediately post-intervention. Hence, this study contributes valuable CBT data on Malaysian orphaned adolescents.

The pre-intervention scores of negative automatic thoughts in this study significantly predicted the intervention outcome for the pre-post intervention change score of depression. This result not only supports the CBT treatment model for adolescent depression but also contributes to research findings in adolescent samples regarding the amount of depression recovery before and after CBT intervention.

This study found that the pre-intervention score of self-esteem did not predict the immediate post-intervention outcome of depression but did predict the pre-post intervention change score of depression. This outcome is consistent with Waite et al. (17), who observed that low self-esteem predicted poorer outcomes in psychotherapy for depression. Despite a high mean pre-intervention self-esteem score in this study, Sowislo and Orth (39) note that while self-esteem and depression are often linked, they are not consistently strong predictors of each other. Waite et al. (17) reiterated that depression may lower self-esteem, while others propose that low self-esteem predisposes depression. Thus, it is possible that the increase in self-esteem from pre- to post-intervention in this study enhanced its predictive value for depression. However, the scarcity of research in this specific area limits the generalization of these study results.

A mildly elevated pre-intervention score of anger at baseline did not predict the intervention outcome immediately post-intervention or the pre-post intervention change score of depression. Limited existing studies on the predictive abilities of CBT response for anger treatment mean that this study’s findings offer only preliminary evidence. Moreover, anger management was not a specific focus of the intervention in this study, although emotional regulation was addressed more broadly. It is also possible that most participants had only mildly elevated anger levels, and the decrease in anger might have been too little to yield a significant effect. Notably, only dysfunctional anger predicted depression as reported by Fernandez and Johnson (16).

Pre-intervention levels of depression, anxiety, and self-esteem significantly predicted immediate post-intervention anxiety outcomes, whereas negative automatic thoughts and anger were not significant predictors.

This aligns with evidence that baseline emotional and functional status is an important determinant of CBT response. For example, anxiety sensitivity has been identified as a key risk factor for both the development and persistence of anxiety symptoms (40), while lower baseline severity and higher global functioning have been associated with better CBT outcomes (10). Similarly, Hill et al. (18) reported greater symptom reduction in adolescents with lower initial anxiety levels following CBT. Notably, although participants in the present study showed moderate baseline anxiety, pre-intervention severity still significantly predicted post-treatment outcomes.

The pre-intervention anxiety score significantly predicted the intervention outcome for the pre-post intervention change score of depression, whereas the pre-intervention depression score did not significantly predict the pre-post intervention change score of anxiety. Seligman and Ollendick (41) suggested that anxiety could be a risk factor for affective disorders like depression, noting that individuals with anxiety may develop depression, but not necessarily vice versa. Clark and Beck (42) also indicated that anxiety disorders often precede depressive disorders rather than the reverse. Furthermore, Liber et al. (43) found that youths with comorbid anxiety and depression were more likely to maintain their primary anxiety diagnosis following CBT. Similarly, Hill et al. (18) mentioned that comorbidity between externalizing and internalizing disorders led to poorer predictions of CBT treatment outcomes for anxiety disorders. Therefore, past literature suggests that while pre-intervention anxiety scores may predict the extent of recovery before and after the gCBT intervention for depression, pre-intervention depression scores may not predict the same for anxiety recovery after the gCBT intervention.

Furthermore, in this study, the pre-intervention score of negative automatic thoughts did not predict the intervention outcome of anxiety immediately post-intervention, possibly due to internalizing comorbidity within anxiety, leading to poor prognosis. Despite being statistically insignificant (*p* = .05), the predictive ability of the pre-intervention score of negative automatic thoughts to predict the pre-post intervention change score of anxiety was very close to significant. This suggests that the gCBT was effective despite the high pre-intervention score of negative automatic thoughts. Muris et al. (15) discovered that diminished negative automatic thoughts and enhanced anxiety control were predictive of the efficacy of gCBT for anxious adolescents. Despite the high pre-intervention score of negative automatic thoughts at baseline in this study, it has a relatively minor influence on the amount of recovery before and after gCBT intervention for anxiety.

Also, in this study, a high pre-intervention score of self-esteem predicted the intervention outcome of anxiety immediately post-intervention and the pre-post intervention change score of anxiety. This finding aligns with Sowislo and Orth’s (39) observation that self-esteem and anxiety have an equal ability to predict each other. However, interpreting the predictive ability of self-esteem for depression/anxiety, and vice versa, should be approached cautiously due to the possibility of reversed causality between self-esteem and depression/anxiety. Some studies suggest that depression and anxiety can lower self-esteem, while others indicate that low self-esteem may predispose individuals to depression and anxiety (17). Keane and Loades (44) also noted a positive association between low self-esteem and depression in adolescents, particularly those with comorbid depression and anxiety.

Conversely, in this study, the mildly elevated pre-intervention score of anger at baseline did not significantly predict the intervention outcomes of anxiety immediately post-intervention or for the pre-post intervention change score of anxiety. This finding provides only preliminary evidence, suggesting that the decrement in anger may have been insufficient to produce a significant effect. Fernandez and Johnson (16) noted that only dysfunctional anger predicted anxiety, indicating a need for further research to generalise this finding.

Overall, the GLMM results show mixed evidence across outcomes. While one model demonstrated a strong and robust effect with high statistical significance and a large effect size, several other models showed statistically significant or borderline *p*-values accompanied by confidence intervals that included the null value. Effect sizes ranged from small to medium, suggesting that although some effects may be practically meaningful, the precision of estimates was inconsistent. These findings indicate that the observed associations should be interpreted with caution.

## 5 Conclusion

Overall, the present study demonstrated that the type of intervention was the better predictor of intervention outcomes for depression and anxiety, both at immediate post-intervention and in pre-post intervention change scores, compared to other predictors. Although this was a short-term predictor analysis, the randomised controlled trial included follow-up assessments at 1-, 3-, and 6-month post-intervention, with gCBT demonstrating sustained effectiveness for depression and anxiety up to 6 months after treatment (22). Adolescents in the gCBT group showed greater improvement than those in the waitlist control group, supporting the effectiveness of CBT-based interventions for emotional difficulties. However, follow-up data were not included in the present predictor analysis due to substantial attrition, which reduced statistical power and introduced potential bias. This study, therefore, focused on immediate post-intervention outcomes using the most complete dataset.

Psychological variables, particularly baseline levels of depression, anxiety, self-esteem, and negative automatic thoughts, showed stronger predictive abilities than most sociodemographic variables. Among the sociodemographic variables examined, only age significantly predicted intervention outcomes, while gender, type of orphanhood, and history of counselling attendance did not significantly influence treatment responsiveness.

The findings also provide important clinical implications. The significant predictive role of psychological variables highlights the importance of conducting comprehensive baseline psychological assessments before intervention. Identifying adolescents with elevated anxiety, depressive symptoms, maladaptive cognitions, and low self-esteem may help clinicians tailor intervention strategies and improve treatment responsiveness. The findings further suggest that gCBT can be effectively implemented across different adolescent demographic groups, thereby supporting its broader applicability within school, community, and residential care settings in Malaysia.

Importantly, to the best of the researcher’s knowledge, following an extensive, although not exhaustive, review of the literature, this study is among the first in Malaysia to examine predictors of gCBT intervention outcomes among adolescents. As such, the findings contribute meaningful preliminary evidence to the limited body of local literature on adolescent mental health intervention research, particularly within vulnerable populations such as institutionalised adolescents.

Nevertheless, several limitations should be acknowledged. The sample size for the predictor analyses was relatively small, which may have reduced statistical power and limited the generalisability of the findings. Future studies should recruit larger and more diverse samples involving multi-site recruitment and longitudinal follow-up assessments to strengthen the robustness and external validity of the results. Nonetheless, this study was conducted in Malay-operated non-government shelters due to limited accessibility and the small number of government-run shelters available for research participation. This approach also ensured consistency in the language of administration, as multilingual and translation variability across different institutional settings in Malaysia could introduce measurement bias.

Methodologically, the use of the GLMM represents a notable strength of this study. Compared to conventional approaches such as repeated measures ANOVA, correlational analyses, and hierarchical multiple regression, GLMM provides greater flexibility in handling missing data, non-linear distributions, repeated measurements, and contextual variations such as intraclass correlations and unequal time intervals (35, 45). The application of GLMM, therefore, enhances the reliability and validity of the present findings.

A significant discrepancy in prior school counselling attendance among the participants may reflect differences in the type and purpose of earlier help-seeking rather than differences in treatment quality or adequacy. Participants reported that previous counselling was generally brief (often fewer than three sessions) and focused on specific issues such as learning difficulties or religious adherence, rather than structured psychological intervention for the presenting condition. This suggests that prior school counselling history likely represents heterogeneous and limited exposure to support services, which may account for the observed group differences.

This study relied solely on participant self-report measures, which may be subject to response bias. Future studies should incorporate objective behavioural indicators (e.g., school attendance, academic performance, or observational data) to provide a more comprehensive evaluation of intervention effects. Clinical characteristics such as onset, severity, duration, and comorbidity are crucial predictors of treatment outcomes, deserving thorough investigation (46). In addition to prediction research design, conducting moderator and mediator analyses is recommended to assess the relative efficacy of specific therapeutic factors for treatment outcomes. Furthermore, employing structural equation modelling and other advanced data analytics strategies can help extend the theoretical framework of CBT.

Future research should incorporate longer-term follow-up assessments more than 6 months, as extended monitoring is crucial for evaluating the durability of g-CBT treatment gains and for predicting long-term outcomes. Prior studies have similarly emphasised the importance of multi-point follow-up data to understand whether intervention effects are sustained over time (47). This research also can expand to encompass diverse ethnic and minority groups, as well as children aged 12 and below and adults. The research population can also include children and adolescents in clinical settings, specific diagnostic groups, and transdiagnostic groups with comorbid disorders.

Evidence from the COVID-19 period shows that adolescents experienced significantly increased anxiety and depressive symptoms during large-scale disruptive events (e.g., Samji et al., 2022). This study was conducted in 2014–2015, before the COVID-19 pandemic; therefore, pandemic-related stressors did not influence participants’ baseline functioning. However, major life events can shape psychological symptoms in young people. Given that our participants were orphans, other contextual or personal stressors (e.g., bereavement, instability, institutional transitions) may have influenced their emotional functioning but were not systematically measured. Future studies should assess life-event exposure to better understand potential confounding influences.

## Data Availability

The raw data supporting the conclusions of this article will be made available by the authors, without undue reservation.
